# Conservation of the unusual dimeric JmjC fold of JMJD7 from *Drosophila melanogaster* to humans

**DOI:** 10.1038/s41598-022-10028-y

**Published:** 2022-04-11

**Authors:** Rasheduzzaman Chowdhury, Martine I. Abboud, James Wiley, Anthony Tumber, Suzana Markolovic, Christopher J. Schofield

**Affiliations:** Chemistry Research Laboratory, Department of Chemistry and the Ineos Oxford Institute for Antimicrobial Research, University of Oxford, Mansfield Road, Oxford, OX1 3TA UK

**Keywords:** Biochemistry, Chemical biology, Drug discovery, Structural biology

## Abstract

The JmjC family of 2-oxoglutarate dependent oxygenases catalyse a range of hydroxylation and demethylation reactions in humans and other animals. Jumonji domain-containing 7 (JMJD7) is a JmjC (*3S*)*-*lysyl-hydroxylase that catalyses the modification of Developmentally Regulated GTP Binding Proteins 1 and 2 (DRG1 and 2); JMJD7 has also been reported to have histone endopeptidase activity. Here we report biophysical and biochemical studies on JMJD7 from *Drosophila melanogaster* (dmJMJD7). Notably, crystallographic analyses reveal that the unusual dimerization mode of JMJD7, which involves interactions between both the N- and C-terminal regions of both dmJMJD7 monomers and disulfide formation, is conserved in human JMJD7 (hsJMJD7). The results further support the assignment of JMJD7 as a lysyl hydroxylase and will help enable the development of selective inhibitors for it and other JmjC oxygenases.

## Introduction

2-Oxoglutarate (2OG) and Fe(II) dependent oxygenases catalyse hydroxylations, demethylations and other two-electron oxidations on a range of substrates, including proteins, nucleic acids, small molecules, and lipids in humans and other animals^1–3^. In total, there are 60–70 human 2OG oxygenases, some of which have been developed as drug targets. Notably, hypoxia inducible factor (HIF) prolyl hydroxylase (PHD1-3) inhibitors have been approved for the treatment of anaemia in chronic kidney diseases, because transcription of the erythropoietin gene is upregulated by HIF^4,5^. Catalysis by the PHDs signals for proteasome regulated degradation of HIF-α isoforms^5^. The PHDs belong to a structural subfamily of prolyl hydroxylases which extends from humans to some bacteria^5^. A second type of human 2OG oxygenase, Factor Inhibiting HIF (FIH), also negatively regulates HIF activity by reducing its affinity for transcription promoting histone acetyl transferases^5,6^. FIH also plays a role in the context-dependent regulation of the sets of HIF target genes that are upregulated in response to hypoxia^6^. Crystallographic analyses have revealed that FIH belongs to the JmjC subfamily of 2OG oxygenases^2,3^. Subsequently, the largest identified family of histone N^ε^-methyl lysine demethylases (KDMs) was also shown to have a JmjC type fold (JmjC KDMs)^3,7^.

Like all other identified 2OG oxygenases, both the PHDs and the JmjC KDMs have a core distorted double-stranded β-helix (or cupin) fold^2,3^. Different subfamilies of 2OG oxygenases are characterised by modifications and additions to this core DSBH fold. Within the set of JmjC 2OG oxygenases, two broad subfamilies have been identified based on structural similarities and the types of reactions catalysed^2,3^. One of these is the JmjC KDMs, some of which also have N-methyl arginine demethylase (RDM) activity^8^, which typically (but not always) are monomeric and which normally have multiple other domains, at least some of which are often involved in chromatin binding^2,3^. By contrast, the JmjC hydroxylases usually do not have multiple domains and, at least most of them do not have KDM or RDM activity^2,3,7^. As exemplified by FIH, the JmjC hydroxylases typically catalyse hydroxylation of methylene groups on protein residue side chains to give stable alcohol products, though at least one ‘JmjC hydroxylase’ modifies RNA^2,3^.

**Figure 1:**
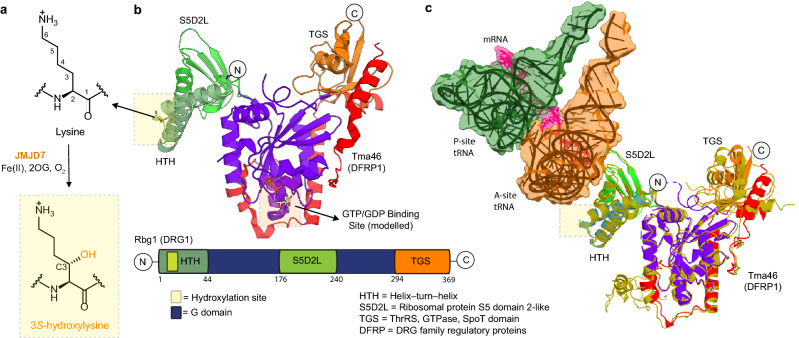
Lysyl hydroxylation sites in DRG1 and 2. (**A**) JMJD7-catalysed (3*S*)-lysyl hydroxylation. (**B**) Views from a crystal structure of Rbg1.Tma46 complex (the yeast homologue of human DRG1.DFRP1, PDB: 4A9A) highlighting the hydroxylation site. All DRG orthologues, including Rbg1, contain three additional domains (HTH, S5D2L and TGS) surrounding the GTPase domain. JMJD7 hydroxylates a highly conserved lysine residue at the apex of the DRG HTH domain (boxed yellow; see Figs. S1b and S2a for an alignment of DRG orthologues). (**C**) Views from the cryo-EM structure of yeast 80S ribosome bound to Rbg1.Tma46 complex (PDB: 7RR5, EMD-24652) showing molecular interactions between Rbg1 and the ribosomal A-site tRNA (for simplicity, the rest of the ribosome is not shown)^14^. The figure shows superimposed views of the Rbg1.Tma46 complexes in the free (PDB: 4A9A, colour coded as in (**B**) and ribosome-bound (PDB: 7RR5, golden-yellow) forms revealing conformational changes, particularly involving the Rbg1-TGS domain and Tma46 on ribosome binding. Given that the target lysine sidechain is in close contact with A-site tRNA, hydroxylation may affect interactions with the ribosome.

DRG1 and 2 are multidomain Translation Factor (TRAFAC) class GTPases that are present in archaea, eukaryotes and plants and have roles in protein synthesis, signal transduction, cell motility, and transport^9,10^. DRG1 and 2 contain a guanine nucleotide-binding (G) domain, which is flanked by HTH (helix-turn-helix) and TGS (ThrRS, GTPase, SpoT) domains that can recruit auxiliary protein(s) for efficient GTP hydrolysis^11,12^ (Fig. 1). Except for their canonical G-domain, the additional domains of DRG1 and DRG2 do not share sequence similarities with other known GTPases, and their functions are in general unclear^11,12^.

DRG1 was first identified in the developing murine brain and frog embryos and was shown to play a role in animal development^13^. The yeast orthologue of DRG1 (Rbg1) is reported to directly interact with the 80S ribosome (Fig. 1c) and to promote translational efficiency by suppressing ribosomal pausing^14^. Human DRG1 is involved in spindle dynamics by promoting polymerisation, binding, and stabilisation of microtubules^15^. The role of DRG1 in spindle checkpoint assembly makes it potentially pro-oncogenic^16^. However, the observations that knockdown of the *DRG1/2* genes in human cell lines^17^, or deletion of their counterparts in yeast^18^, have no clear phenotypes suggests that the roles of the GTPase activity of DRG1/2 may be context-dependent, requiring specific conditions, e.g. post-translational modifications (PTMs), to ‘switch on’ their regulatory functions. Indeed, phosphorylation of a threonine (T100) located on the GTP binding domain switch I loop in DRG1 substantially promotes DRG1 catalysed GTP hydrolysis^12,19^.

We have reported that (3*S*)*-*lysyl hydroxylation of both human DRG1 (at K22) and DRG2 (at K21) is catalysed by the 2OG oxygenase JmjC (Jumonji-C) domain-containing 7 (JMJD7) (Fig. 1)^20^. Although JMJD7 catalysed lysyl-hydroxylation of DRG1 had no apparent direct effect on its GTPase activity, the hydroxylated DRG1 displayed a substantially reduced sensitivity to proteases suggesting a potential role for lysyl hydroxylation in regulating the stability of DRG proteins in cells^20^. Interestingly, JMJD7 has also been reported to have histone endopeptidase activity^21^. Mutations to the human JMJD7 gene have been correlated with autism^22^, intellectual disability^23^ and cancer^24^.

The finding that human JMJD7 (hsJMJD7) catalyses lysyl hydroxylation of DRG1 and 2 expands the interface between oxygen and the regulation of protein function^2,3^. From a biophysical perspective, crystallographic analyses of hsJMJD7^20^ were particularly interesting because they revealed an unprecedented mode of 2OG oxygenase/JmjC protein dimerization, involving both the N- and C-terminal regions of both monomers, as well as disulfide bond formation. Here we describe biochemical, and crystallography studies on dmJMJD7. The results provide evidence for the conservation of function of JMJD7 as DRG1 and 2 hydroxylases and, importantly, of the unusual dimerization mode of JMJD7.

## Results

### Substrate selectivity of dmJMJD7

A cross-genomic phylogenetic analysis of hsJMJD7 sequences has identified hsJMJD7 orthologues in a range of eukaryotes including plants, including in *Mus musculus* (90% sequence identity), *Danio rerio* (66%), *Drosophila melanogaster* (45%) and *Glycine max* (40%) (Fig. S1a). *D. melanogaster* is of particular interest because of its utility for studying diseases, in part, owing to its short generation time, the efficiency of genetic manipulations, and the high sequence similarity between many human and *D. melanogaster* proteins^25^. The regulation of cell size in the *Drosophila* wing upon dmJMJD7 knockdown suggests its phenotype may be related to hydroxylation of a DRG-like protein that is involved in protein synthesis and cell growth, at least in humans^20^. Unlike higher animals, the *D. melanogaster* genome contains a single copy of a DRG-like protein, annotated as a Guanylate Binding Protein (GBP) that has > 80% sequence identity to human DRG1/2 (Fig. S1b).

We produced recombinant dmJMJD7 in *Escherichia coli* and tested the purified enzyme for its ability to hydroxylate *Drosophila* GBP as well as human DRG1 and 2 peptide substrate fragments that are efficiently hydroxylated by hsJMJD7^20^. The results of mass spectrometric (MS) assays reveal that dmJMJD7 efficiently hydroxylates *D. melanogaster* GBP and both human DRG1 and DRG2 peptide fragments in the presence of Fe(II), 2OG and ascorbate, though the latter is not essential for JMJD7 catalysis. The MS fragmentation analyses of hydroxylated GBP and DRG1/2 show that dmJMJD7 catalysed hydroxylation occurs at the same DRG1 and DRG 2 lysine-residues (K22 for DRG1 and K21 for DRG2) as the hsJMJD7 catalysed reactions and occurs on the analogous lysine residue in the *Drosophila* GBP peptide (K22) (Figs. S2, S3). Given the observation that human DRG1/2 peptides are hydroxylated by both the hsJMJD7 and dmJMJD7 enzymes, the inverse was of interest, i.e. testing GBP with hsJMJD7. Hydroxylation assays confirmed that hsJMJD7 catalyses hydroxylation of GBP (Fig. S2c), implying conservation of JMJD7 function in humans and *D. melanogaster,* in support of prior cellular results^20^.

### Crystal structures of dmJMJD7

The observation that both hsJMJD7 and dmJMJD7 catalyse DRG/GBP hydroxylation raises the question as to their evolutionary relationship from a structural perspective, given that they share only ~ 45% sequence identity (Fig. S1a)^20^. We therefore carried out crystallographic analyses on dmJMJD7. We obtained dmJMJD7 crystals of His_6_-dmJMJD7.Mn complexed with its co-substrate 2OG or co-product succinate; catalytically inert Mn(II) was used instead of the cofactor Fe(II) to block catalysis under aerobic conditions, as was done for hsJMJD7^20^. The His_6_-dmJMJD7.Mn(II) 2OG complex structure was solved by molecular replacement using PHASER^26^ using a hsJMJD7 crystal structure (PDB: 5NFO) with two dmJMJD7 molecules per asymmetric unit in the *P*2_1_ space group. All other dmJMJD7.ligand structures were subsequently solved using the His_6_-dmJMJD7.Mn.2OG structure (PDB: 7YXG) as the initial search model.

**Figure 2:**
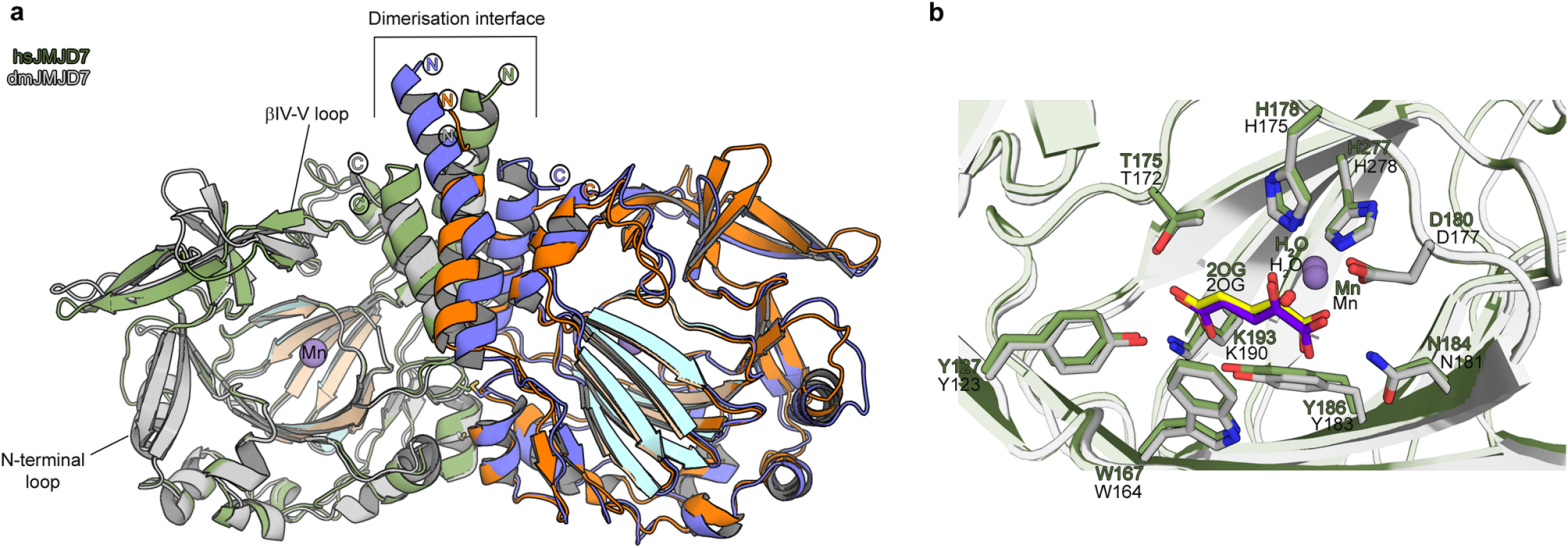
Comparison of the crystal structures of dmJMJD7 and hsJMJD7. (**A**) Except for the dimerization interfaces, the overall structures of dmJMJD7 and hsJMJD7 oxygenases adopt similar folds, despite only 45% sequence conservation. The core distorted DSBH fold is highlighted. (**B**) Views from the active sites of dmJMJD7 and hsJMJD7 showing very similar metal ion (Mn in place of Fe) coordination and co-substrate (2OG) binding modes.

**Figure 3:**
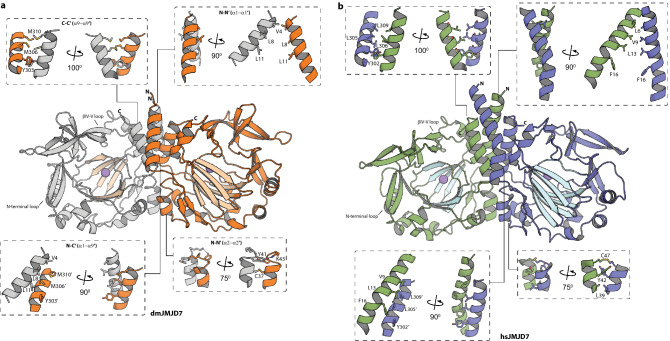
Both dmJMJD7 and hsJMJD7 are dimeric 2OG-oxygenases. Structural analysis of the oligomerization domains of *Drosophila* (**A**) and human (**B**) JMJD7 reveals a conserved role for α-helices in oligomerization between monomers, which is reminiscent of dimerization in FIH and TYW5^2,3^, though the nature of the interactions differs. While FIH and TYW5 employ C-terminally located α-helices for dimerizations, JMJD7 dimerizes via a mode involving both N- (α1 and α2) and C- (α9) terminal α-helical regions (see text for details).

The His_6_-dmJMJD7.Mn structures reveal that the overall fold of dmJMJD7 is very similar to that of hsJMJD7 (root mean square deviation of 1.3 Å for Cα atoms of > 90% residues, Fig. 2a). Like hsJMJD7, dmJMJD7 forms a homodimer with each 316-amino-acid monomer possessing an N-terminal helix-loop-helix-fold domain (aa 1–42) that forms part of the dimerization interface, a core distorted double-stranded β-helix (DSBH, aa 160–294) fold comprising of eight parallel β-strands and an α-helical C-terminal domain (aa 296–310) which is also involved in dimerization (Fig. 3). Despite the overall similarities of the hsJMJD7 and dmJMJD7 folds, there are differences in their secondary structural elements: the length of both N- and C-terminal helices (α1 and α11) are shorter by two helical turns in dmJMJD7 compared to hsJMJD7; the DSBH IV-V loop, which is likely involved in DRG1/2 substrate binding and an N-terminal loop prior to DSBH I are extended in dmJMJD7 by 4 to 3 residues, respectively, compared to hsJMJD7 (Fig. 3a).

Crystallographic analysis of the His_6_-hsJMJD7.Mn.2OG complex (PDB: 5NFO) revealed an unprecedented dimerization mode, with interactions between monomers involving both the N- and C-terminal regions; the hsJMJD7 dimer is further stabilised by a disulphide link between two Cys47 residues (located on α2) from each monomer (Fig. 3b)^20^. As with hsJMJD7, the dmJMJD7 homodimer is formed by both N- and C-terminally located helical bundles that pack closely in a head-to-tail manner (Fig. 3a). The dmJMJD7 dimerization interface for the N–C–C′–N′ (α_1_-α_1_′, α_1_-α_9_′, and α_9_–α_9_′) and N–N′ (α_2_–α_2_′) dimerization regions has a combined buried surface area of ~ 1756 Å^2^, compared to ~ 1700 Å^2^ for hsJMJD7 (Fig. 3a). Two methionine-residues (Met306 and Met310 on α10) that face towards the protein interior and a Tyr303 (α10) from each monomer form the hydrophobic core of the C–C′ symmetrical dimer interface. The C–C′ dimer interface is flanked on each side by N-terminal residues, i.e. two leucines (Leu8 and Leu11 on α1) and a valine (Val4 on α1) which are positioned to make hydrophobic contacts with the analogous residues in the other monomer. The N–N′ (α_2_–α_2_′) dimerization interface involves hydrophobic (Leu34–Leu34′ on α_2_) and electrostatic (Tyr41-Lys43′ on α_2_) interactions between the monomers. Notably, the two monomers are covalently linked by a disulfide bond (Cys37–Cys37′ on α_2_), as observed in crystallographic analysis of hsJMJD7^20^. The buried surface area and complementarity of residues at the dimer interface is significant enough for JMJD7 dimerization as observed in solution^20^.

Consistent with a higher degree of conservation of JMJD7 residues located on the DSBH core fold (~ 68%) than elsewhere (Fig. S1a), both hsJMJD7 and dmJMJD7 share almost identical active site features, an observation in accord with our biochemical observations showing their substrates are interchangeable. Like hsJMJD7, dmJMJD7 has an active site metal ion (Mn substituting for Fe) that is octahedrally coordinated by the 2OG oxalyl group, a water molecule, and the highly (but not universally^27^) conserved metal-binding triad of residues (H175, D177, and H278) (Fig. 2b). The 2OG oxoacid coordinates the metal ion with its 2-oxo carbonyl oxygen ligating *trans* to D177 and one of its C1 carboxylate oxygens ligating *trans* to H175; the other C1 carboxylate oxygen is positioned to form an H-bond with the sidechain amide of N181. The 2OG C5 carboxylate is positioned to form electrostatic/ H-bond interactions with the side-chains of four dmJMJD7 residues, Lys190 (βIV), Tyr123 (non-DSBH aa), Thr172 (βII), and Tyr183 (βIII). The conservation of 2OG C5 carboxylate binding mode of hsJMJD7 in dmJMD7 is particularly notable since this feature is uniquely characteristic of JMJD7 amongst the structurally characterised 2OG oxygenases (Fig. S4).

### dmJMJD7 inhibitor complex structures

**Figure 4:**
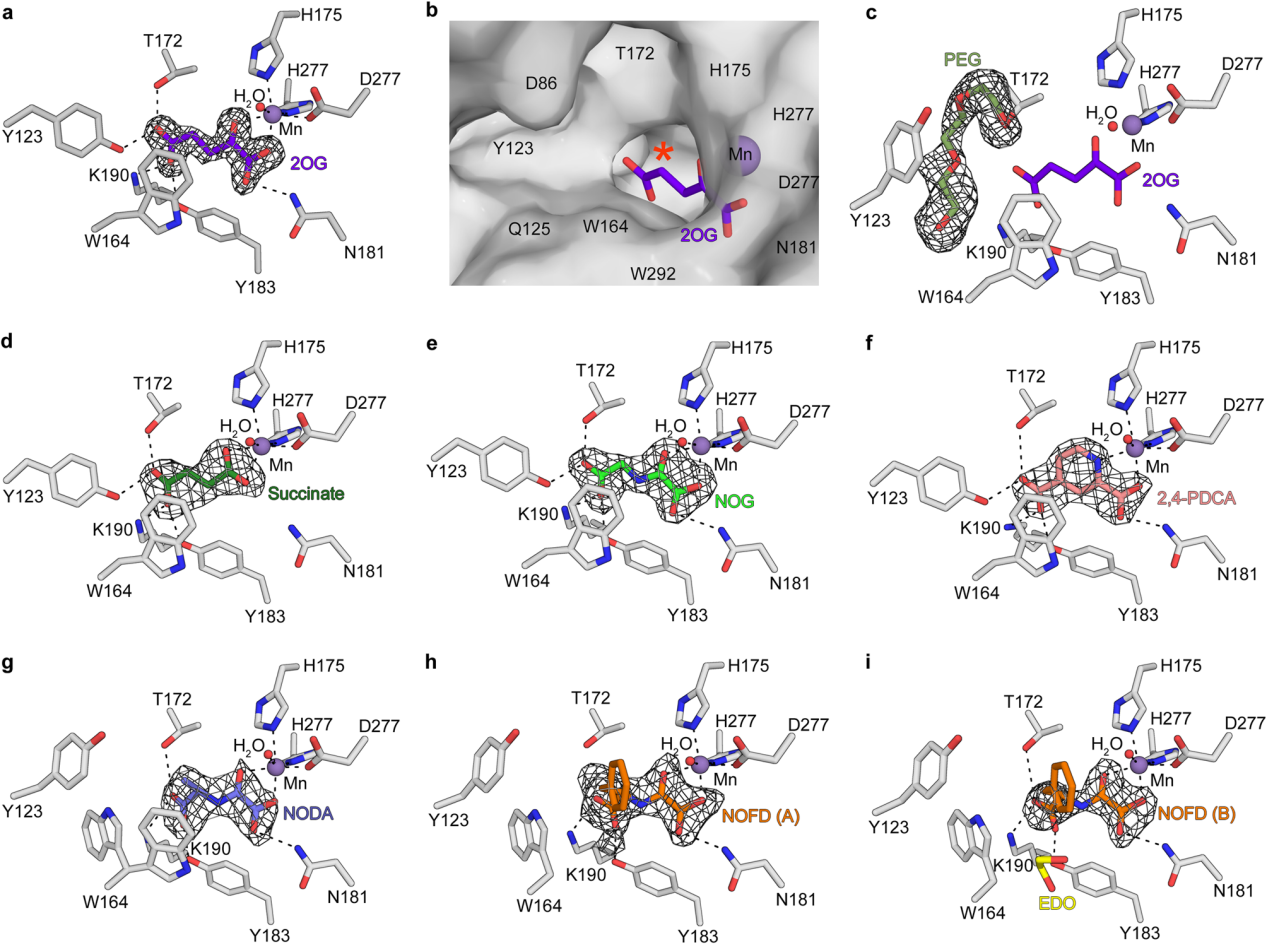
Views from the crystal structures of dmJMJD7.MnII complexes. Active site views from dmJMJD7.2OG (**A**,**B**), 2OG.PEG (**C**), succinate (**D**), NOG (**E**), 2,4-PDCA (**F**), N-oxalyl-*D*-alanine (NODA) (**G**), N-oxalyl-*D*-phenylalanine (NOFD, **H**,**I**) complexes. Meshes represent Fo-Fc OMIT maps contoured to 3σ.

2OG oxygenase inhibition is presently of considerable interest from a medicinal chemistry perspective, with (HIF) prolyl hydroxylases (PHD1-3) inhibitors being approved for the treatment of anaemia^4,5^ and JmjC demethylases being pursued for the treatment of cancer^28^. Most reported 2OG oxygenase inhibitors bind to the active site Fe(II) and compete with 2OG. Comprehensive knowledge of how such 2OG mimetics bind to different human 2OG oxygenases is of interest both with respect to optimising potency for medicinal targets and small-molecule probes of oxygenase function as well as minimising off-target inhibition. We thus attempted to obtain structures of hsJMJD7 and dmJMJD7 with 2OG analogues and found that dmJMJD7 was particularly amenable to such studies. We obtained His_6_-dmJMJD7.Mn crystal structures in complex with the near isosteric 2OG analogue N-oxalylglycine (IC_50_ 0.3 μM, as shown by a solid-phase extraction coupled to MS assays using DRG1 (aa 16–40) peptide substrate), N-oxalyl-*D*-alanine (IC_50_ 4.14 μM), N-oxalyl-*D*-phenylalanine, and pyridine-2,4-dicarboxylate (2,4-PDCA, IC_50_ 1.34 μM) (Fig. 4).

The structures reveal that all four compounds bind at the dmJMJD7 2OG binding site by bidentate coordination of the metal ion *trans* to H175 and D177 and by interacting with the 2OG C5 carboxylate binding residues in a closely analogous bidentate manner to that observed for 2OG itself (Fig. 4). Notably, the methyl group of N-oxalyl-*D*-alanine and benzyl group of N-oxalyl-*D*-phenylalanine project into an active site region that, in the absence of these ligands, can also be occupied by ethylene glycol (used for cryo-protection) or a polyethylene glycol fragment (used for crystallisation) located within 4.0 Å of the 2OG C4 methylene group (Fig. 4c). The binding of N-oxalyl-*D*-alanine and N-oxalyl-*D*-phenylalanine to the JMJD7 active site is interesting given both these compounds are inhibitors of the related JmjC enzyme, FIH^29^. Indeed, comparison of the dmJMJD7 and FIH^29^ crystal structures in complex with N-oxalyl-*D*-phenylalanine reveals that the benzyl groups adopt similar geometries in the two active sites (Fig. S5). Thus, the results presented here suggest that care should be taken in the cellular use of N-oxalyl-*D*-phenylalanine as a selective FIH inhibitor since it may also inhibit JMJD7.

Comparison of the dmJMJD7 crystal structures reveals a flexible region (aa 123–138) located N-terminal to the DSBH β-strands, which normally forms a loop around the dmJMJD7 active site and is likely involved in induced-fit during substrate binding. Compared to that observed in the dmJMJD7.Mn.2OG/succinate/N-oxalylglycine/2,4-PDCA structures, this loop adopts a different conformation and forms a non-DSBH β-strand extending the major DSBH β-sheet in the N-oxalyl-*D*-alanine and N-oxalyl-*D*-phenylalanine complexes. Close inspection of the active site in the different complex structures reveals that binding of the N-oxalyl-*D*-alanine or N-oxalyl-*D*-phenylalanine inhibitors induces conformational changes of the Trp164 and Tyr123 sidechains (which are rotated by ~ 90 and 80° relative to the 2OG/succinate/N-oxalylglycine structures). Notably, in the 2OG/succinate/N-oxalylglycine complexes, Trp164 is positioned to form a stacking interaction with the methylene groups of 2OG/succinate/N-oxalylglycine; the C4-methylene substitution in N-oxalyl-*D*-alanine or N-oxalyl-*D*-phenylalanine compared to N-oxalylglycine prevents this interaction. Tyr123 is one of four dmJMJD7 residues positioned to form H-bonds with the 2OG/succinate/N-oxalylglycine C5-carboxylate (Figs. 4 and S4). The introduction of the benzyl group in N-oxalyl-*D*-phenylalanine also apparently distorts the ligand binding at the C5-carboxylate site with respect to that of dmJMJD7.2OG/NOG complexes (Fig. 4). Thus, it appears that binding of 2OG/ inhibitory 2OG mimetics at the JMJD7 active site likely induces coordinated motions involving at least several residues, as also appears to be the case for the HIF prolyl hydroxylase PHD2^30–32^.

## Discussion

Protein hydroxylation and N-methyl demethylation via hydroxylation, as catalysed by 2OG oxygenases, have emerged as post-translational modifications of fundamental importance in eukaryotic biology^2,3,7^. 2OG oxygenase catalysed hydroxylation to give stable alcohol products has been identified on the side chains of multiple protein residues, including prolyl, asparaginyl, aspartyl, lysyl, arginyl, and histidinyl residues in eukaryotic proteins. The enzymes catalysing such reactions appear to operate with varying degrees of selectivity, at least in cellular and in vitro studies, with FIH, AspH, and Jumonji domain-containing 6 (JMJD6) being rather promiscuous. By contrast, other protein hydroxylases appear to be much more selective, e.g. the HIF-α hydroxylating PHDs^2,33,34^. Lysine-residues are particularly notable for their propensity to undergo 2OG oxygenase catalysed modifications. In addition to N^ε^-methyl group demethylation, the methylene groups of lysyl side chains can be modified by hydroxylation at the C5, C4, and, as catalysed by JMJD7, C3 positions, giving rise to independent PTMs^2,3^. Lysyl-residue hydroxylation can occur to give (5*R)-*hydroxylated products as catalysed by the procollagen lysyl hydroxylases (PLODs)^35^, (5*S*)*-*hydroxylated products as catalysed by JMJD6^36,37^, and a C4 hydroxylated product as catalysed by Jumonji domain-containing 4 (JMJD4)^38^.

2OG oxygenases have established roles in regulating chromatin function/ transcription, e.g. the JmjC KDMs catalyse N^ε^-methyl lysine demethylation, and the TET enzymes catalyse 5-methylcytosine modifications^39^. The activity of the hypoxia-inducible transcription factor is tightly controlled by prolyl- and asparaginyl-hydroxylases^34^. 2OG oxygenases are also involved in the regulation of RNA; thus, JMJD6 is proposed to be involved in the regulation of mRNA splicing by catalysing hydroxylation of splicing factors^40^, and in animals, the fat mass and obesity protein (FTO) and AlkB homologue 5 (ALKBH5) are N^6^-methyladenine hydroxylases/demethylases, and TWY5 and ALKBH8 hydroxylate the anticodon loops of some tRNAs^2,28,39^. Recent work suggests that the translational machinery is also a major target of 2OG oxygenases. MYC-induced nuclear antigen (MINA53/ RIOX2) and nucleolar protein 66 (NO66/ RIOX1) are closely related JmjC oxygenases that catalyse histidinyl hydroxylation of ribosomal proteins Rpl27a and Rpl8, respectively^41,42^. OGFOD1 is a prolyl hydroxylase of Rps23 that controls decoding during translation^43^. JMJD4 regulates translational termination via lysyl hydroxylation of the termination translation factor, eukaryotic release factor 1 (eRF1)^38^.

It has been reported that hsJMJD7, a JmjC 2OG-oxygenase catalysing (3*S*)-lysyl hydroxylation of both human DRG1 and 2, plays essential roles in cellular processes, including protein synthesis (amongst others)^20^. The unique (to date) ability of JMJD7 to catalyse lysyl C3 hydroxylation coupled with its role in regulating protein synthesis means JMJD7 is a JmjC enzyme of particular interest. The *JMJD7* gene, located on human chromosome 15, can form a naturally occurring read-through transcript with the neighbouring PLA2G4B gene to encode a JMJD7-PLA2G4B fusion protein overexpressed in cancers, including breast, prostate, and thyroid cancer^24^. JMJD7 variants have been linked to patients with severe intellectual disabilities (M160V)^23^ and autism (R260C)^22^. These links to cancer and autism make JMJD7 a compelling enzyme to study from the perspective of linking oxygenase activity to physiology and disease, including by using appropriate models to study JMJD7 function at an organismal level.

Our results provide direct evidence that dmJMJD7 catalyses oxygen-dependent hydroxylation of human DRG (1 and 2) and their counterpart in *Drosophila,* i.e. GBP, thus supporting evidence for the involvement of JMJD7 in the regulation of cell size in *D. melanogaster*^20^. The biochemical assay data show a high degree of similarity between the hydroxylations performed by hsJMJD7 and dmJMJD7, as both the hydroxylation reactions they perform, the residues modified, and the efficiency of their hydroxylations are the same/ similar. Despite their relatively low level (45%) of sequence similarity, our substrate selectivity, structural and inhibition results collectively imply remarkable conservation between hsJMJD7 and dmJMJD7 in terms of their catalysis and biochemistry. Thus, *Drosophila* appears to be a suitable model organism for further ‘on-target’ pharmacodynamic (PD) studies for probing JMJD7 function. Indeed, *Drosophila* has been used as a model to study cancer and autism^44,45^, both of which have been linked with JMJD7^22,24^.

Notably, the crystallographic analyses on dmJMJD7 combined with previously reported crystal structures for hsJMJD7^20^ reveal the unusual dimerization mode of JMJD7, which involves interactions between the N- and C-terminal regions of both dmJMJD7 monomers and is likely conserved in all animals making JMJD7. The JMJD7 dimerization mode contrasts with those observed for other JmjC dimers. FIH and TYW5 dimerise via bundles of α-helices located at their C-termini, while MINA53 and NO66 form dimers via interactions between α-helices located between their JmjC and winged-helix domains^3,34,41^. In the case of JMJD7 dimerization, the presence of a conserved disulfide which appears to help link the two monomers, is an unusual feature in 2OG oxygenase oligomers. Given that protein disulfide isomerase is the β-component of the α2β2 collagen prolyl hydroxylase^46^, it is possible that the JMJD7 disulfide is part of a similar mechanism for regulating its activity.

The structural results presented here on JMJD7 complexes will enable research on the development of selective JMJD7 inhibitors, including via computationally guided approaches^47^. To enable studies on the roles of hsJMJD7 and dmJMJD7, we are presently undertaking structure–activity relationship studies employing 2OG mimetics. The combined observations further validate the general utility of 2OG mimetics for use in JMJD7 inhibition and suggest that inhibitor derivatives substituted at the 2OG C4 or analogous position may be of particular utility with JMJD7, as has been the case for the JmjC hydroxylase FIH^29^ and JmjC KDMs^48^. However, the different conformations for the sidechains of key JMJD7 active site residues observed for closely related inhibitors (e.g. N-oxaylglycine and N-oxalyl-*D*-alanine) suggest that care should be taken in assuming conserved binding modes even within the same series, as has also been found to be the case for inhibition of the PHDs where induced conformational changes can manifest on inhibitor (and substrate) binding^30–32^. Thus, it would seem to be important to combine structure/ computationally guided and empirical approaches to achieve potent and selective inhibitors of specific human 2OG oxygenases, such as JMJD7.

## Materials and methods

### Recombinant protein production and purification

The *D. melanogaster JMJD7* gene (encoding full-length dmJMJD7, aa 1–316, Uniprot ID: Q9VU77) was cloned into the pET28a vector (Novagen) by GenScript and used for the production of N-terminally His_6_-tagged dmJMJD7. The required construct was verified by DNA sequencing. Recombinant dmJMJD7 protein was produced in *E. coli* Rosetta™ (DE3) pLysS cells (Novagen). Cells were grown in 2TY media at 37 °C to an OD_600_ of 0.6–0.8. Protein production was induced with 0.5 mM isopropyl β-D-1-thiogalactopyranoside (ITPG); cells were grown overnight at 18 °C. Protein was purified by nickel affinity and size exclusion chromatography in buffer containing 50 mM HEPES pH 7.5, 200 mM NaCl and 5% glycerol. Protein purity was assessed by SDS-PAGE analysis. Highly purified fractions (> 90% by SDS-PAGE analysis) were then pooled and concentrated (25 mg/mL).

### Assays

#### Matrix-assisted laser deionization (MALDI) MS-based hydroxylation assays

Substrate assay mixtures (final volume 20–50 µL in 50 mM HEPES pH 7.5, 200 mM NaCl) containing 50 µM substrate (as defined above) peptide and 10 µM His_6_-dmJMJD7 were incubated for 5–60 min at 37 °C with 200 µM Fe(II), 300 µM 2OG (disodium salt) and 4 mM ascorbate^20^. Reactions were quenched by adding 1% (v/v) formic acid; 1 µL of assay sample was then mixed with 1 µL of α-cyano-4-hydroxycinnamic acid (CHCA) matrix solution (saturated CHCA in 50% acetonitrile, 50% water, 0.1% trifluoroacetic acid) before being spotted onto a Waters 96-spot MALDI plate. The samples were analysed using MALDI-MS on a Waters Micromass™ MALDI micro MX™ instrument in the positive ion reflectron mode.

#### Solid-phase extraction coupled to mass spectrometry (SPE-MS) inhibition assays

Small molecule inhibition of His_6_-dmJMJD7dm activity was assessed by SPE-MS using the DRG1 peptide substrate: ARTQKNKATAHHLGLLKARLAKLRR (synthesized by Severn Biotech, Worcestershire, UK). Enzyme and substrate solutions were prepared in the assay buffer (50 mM Tris. Cl pH7.5, 50 mM NaCl, 1 mM tris(2-carboxyethyl) phosphine (TCEP); reagents were dispensed using a Thermo Multidrop combi dispenser (Thermo Scientific) equipped with a small volume dispensing cassette. All compounds were prepared as a 20 mM stock solution in DMSO. For IC_50_ determinations, an 11-point and threefold serial dilution was prepared in DMSO using an ECHO550 acoustic dispenser (Labcyte); compound serial dilutions were dry dispensed (0.25 µl per well) into a 384-well polypropylene assay microplate (Greiner Bio-One). His_6_-dmJMJD7dm (0.1 µM) was prepared in the assay buffer and 25.0 µl transferred into each well. JMJD7 was pre-incubated with the potential inhibitor for 15 min at room temperature. 25 µl of the assay mixture, consisting of Fe(II) (20 µM), *L*-ascorbic acid (200 µM**)**, 2OG (10 µM**)** and DRG1peptide (10 µM**),** was prepared in the assay buffer. Reactions were initiated by dispensing the DRG1 substrate (25.0 µl) into each well. After 15 min at room temperature, reactions were stopped by adding 5 µl of 10% (v/v) aqueous formic acid. Assay plates were transferred to a RapidFire RF360 robot coupled to an Agilent 6530 Accurate-Mass Quadrupole Time-of-Flight (Q-TOF) mass spectrometer operated in the positive ion mode (Agilent, Wakefield, MA USA).

Samples were aspirated under vacuum for 400 ms, loaded onto a C4 SPE cartridge, and non-volatile buffer salts were removed by washing the cartridge with 0.1% (v/v) formic acid in LCMS grade water at a flow rate of 1.5 ml/min for 5.0 s. The DRG1 peptide was then eluted into the mass spectrometer with 85% (v/v) acetonitrile, 15% (v/v) LCMS grade water, 0.1% (v/v) formic acid at a flow rate of 1.25 ml/min for 5.0 s. The cartridge was re-equilibrated with water for 500 ms. The mass spectrometer was operated with the following parameters: drying gas temperature (350 °C), gas flow rate (12 L/minute), capillary voltage (3500 V), fragmentor voltage (150 V). Ion chromatogram data were extracted for the + 7-charge state for the substrate peptide, and the hydroxylated peptide product and peak area data for extracted ion chromatograms were integrated using RapidFire Integrator software (Agilent, Wakefield, MA, USA). % conversion of the non-hydroxylated peptide to hydroxylated peptide product was calculated using the equation:

$$\% {\text{conversion}} = 100{ } \times {\text{integrated}}\;{\text{product}}/\left( {{\text{integrated}}\;{\text{product}} + {\text{integrated}}\;{\text{substrate}}} \right)$$

IC_50_ data were determined from nonlinear regression curve fit using GraphPad Prism 5.

### Crystallography

Crystals of His_6_-dmJMJD7.Mn(II).ligand complexes (16 mg/mL His_6_-dmJMJD7, 2 mM MnCl_2_, 4 mM ligand) were obtained in sitting drops at 25 °C in buffer conditions containing 0.1 M Bis–Tris pH 5.4, 0.3 M magnesium chloride, 18–23% polyethylene glycol 3350, 0.1 M ammonium acetate (or alternatively, 0.1 M barium chloride) and 0.002 M manganese chloride. Crystals were cryo-protected with 20% ethylene glycol before flash freezing in liquid nitrogen. Data were collected using single crystals at 100 K at the Diamond Light Source (DLS) beamlines and processed using HKL2000^49^. The dmJMJD7.2OG structure was solved by molecular replacement using PHASER^26^ with 2 molecules in the asymmetric unit using a reported hsJMJD7 crystal structure (PDB: 5NFO)^20^. All other dmJMJD7.ligand structures were subsequently solved using the dmJMJD7.2OG structure (PDB: 7YXG) as the initial model. The structures were refined by CNS^50^/ PHENIX^51^ until R_factor_/R_free_ converged with iterative cycles model building using COOT^52^. MOLPROBITY^53^ was used to monitor the geometric quality of the models between refinement cycles. Water molecules were added to peaks (> 1.5σ 2F_o_F_c_) in electron density maps that were within H-bonding distance to protein atoms with a reasonable geometry to form H-bonds.

## Supplementary Information

Supplementary Information.

## Data Availability

GenBank accession codes for the sequences mentioned in this article: Q9VU77 (JMJD7_DROME); P32234 (GTP-binding protein/ 128UP_DROME); P0C870 (JMJD7_HUMAN); Q9Y295 (DRG1_HUMAN); P55039 (DRG2_HUMAN). Coordinates and structure factors for dmJMJD7.2OG (PDB: 7YXG), dmJMJD7.succinate (PDB: 7YXH), dmJMJD7.NOG (PDB: 7YXI), dmJMJD7.2,4-PDCA (PDB: 7YXJ), dmJMJD7.N-oxalyl-*D*-alanine (PDB: 7YXK), and dmJMJD7.N-oxalyl-*D*-phenylalanine (PDB: 7YXL) have been deposited in the RCSB Protein Data Bank and will be released on acceptance of the manuscript. All other data supporting the findings of this study are contained within the published article (and its supplementary information files) or are available from the corresponding author upon reasonable request.
