# Supplementary material for: Conservation of the unusual dimeric JmjC fold of JMJD7 from Drosophila melanogaster to humans
Source: Sci Rep. 2022 Apr 11;12:6065. doi: 10.1038/s41598-022-10028-y (PMC9001643; doi:10.1038/s41598-022-10028-y)
Supplement: Supplementary file 1 — Supplementary Information. [file 41598_2022_10028_MOESM1_ESM.docx]

**Conservation of the unusual dimeric JmjC fold of JMJD7 from *Drosophila melanogaster* to humans**

Rasheduzzaman Chowdhury^1^, Martine I. Abboud^1^, James Wiley^1^, Anthony Tumber^1^, Suzana Markolovic^1^, and Christopher J. Schofield^1*^

^1^Chemistry Research Laboratory, Department of Chemistry and the Ineos Oxford Institute for Antimicrobial Research, Mansfield Road, University of Oxford, Oxford, OX1 3TA U.K.

**Supplementary Figures**

| Figure S1. | Analysis of JMJD7 and DRG sequences. |
| --- | --- |
| Figure S2. | Both dmJMJD7 and hsJMJD7 can catalyse similar cross-hydroxylation reactions. |
| Figure S3. | MS/MS analyses of dmJMJD7 catalysed reaction products |
| Figure S4. | Comparison of the active site views from dmJMJD7.MnII.2OG, dmJMJD7.MnII.2OG.PEG, dmJMJD7.MnII.NOFD and FIH.FeII.NOFD complex structures |
| Figure S5. | 2OG Comparison of 2OG/co-substrate binding in dmJMJD7 and representative JmjC (FIH and KDM4A) and non-JmjC (PHD2) oxygenases. |

**Supplementary Tables**

Table S1. Data collection and refinement statistics of the dmJMJD7.ligand complexes.

**Supplementary References**

**Supplementary Figures**

| **a.** |
| --- |
| **** |
|  |
| **b.** |
| **** |

Figure S1. Analysis of JMJD7 and DRG sequences. (a) Alignment of JMJD7 sequences from different organisms showing metal ion (purple) and co-substrate (2OG, green) binding residues. hsJMJD7 (Uniprot: P0C870), dmJMJD7 (Uniprot: Q9VU77), mmJMJD7 (Uniprot: P0C872), drJMJD7 (Uniprot: Q568J4), gmJMJD7 (NCBI RefSeq: XP_003538923.1) denote JMJD7 from *Homo sapiens*, *Drosophila melanogaster*, *Mus musculus*, *Danio rerio* and *Glycine max*, respectively. (**b**) Sequence comparison of the DRG orthologues showing JMJD7-catalyzed hydroxylation sites in purple. Uniprot database IDs for the select sequences are P39729 (Yeast Rbg1), Q9Y295 (human DRG1), P55039 (human DRG2), and P32234 (*Drosophila* GBP). Secondary structures are drawn based on hsJMJD7 (**a**, PDB: 5NFO) and Rbg1 (**b**, PDB: 4A9A) complex structures.

**
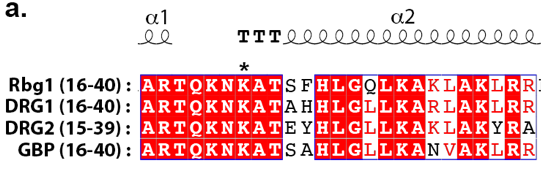
**


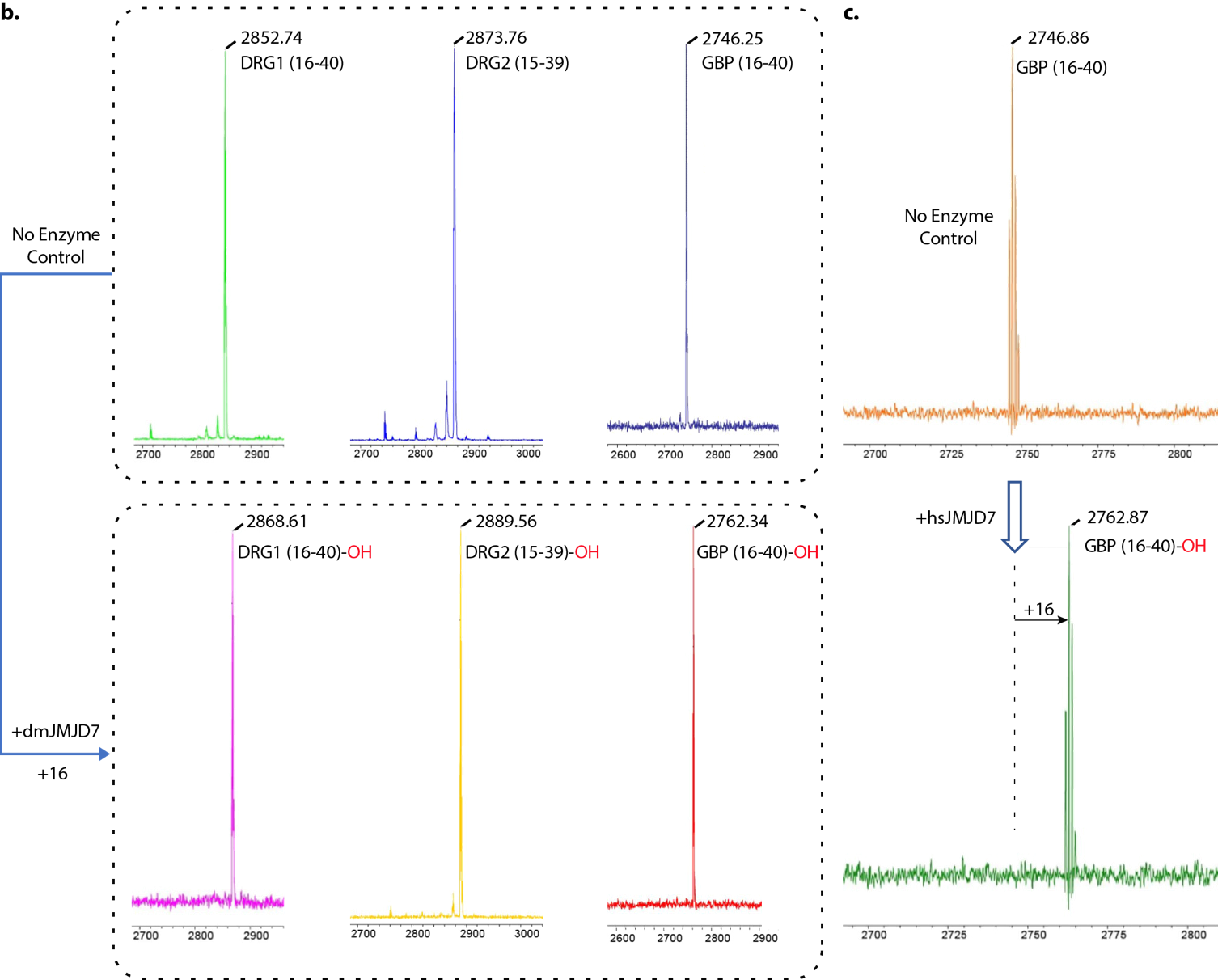


Figure S2. dmJMJD7 and hsJMJD7 can catalyse the hydroxylation of DRG1/GBP. (**a**) Comparison of yeast Rbg1, human DRG1-2, and *Drosophila* GBP peptide sequences shows that the canonical ARTQKNKAT (hydroxylated Lys in red) sequence is highly conserved among different species. Secondary structures of the HTH domains are assigned based on Rbg1.Tma46 complex structure (PDB: 4A9A). (**b**-**c**) MALDI-TOF MS spectra showing dmJMJD7 can catalyse hydroxylation of DRG1-2 and GBP peptides (**b**), while hsJMJD7 can catalyse hydroxylation of *Drosophila* GBP (**c**). Assay mixtures contained 500 µM ascorbate, 100 µM Fe(II), 50 µM peptide and 200 µM 2OG and were incubated for 1 h at 37°C. The +16 peak indicates hydroxylation (+O).

| **a.** |
| --- |
| 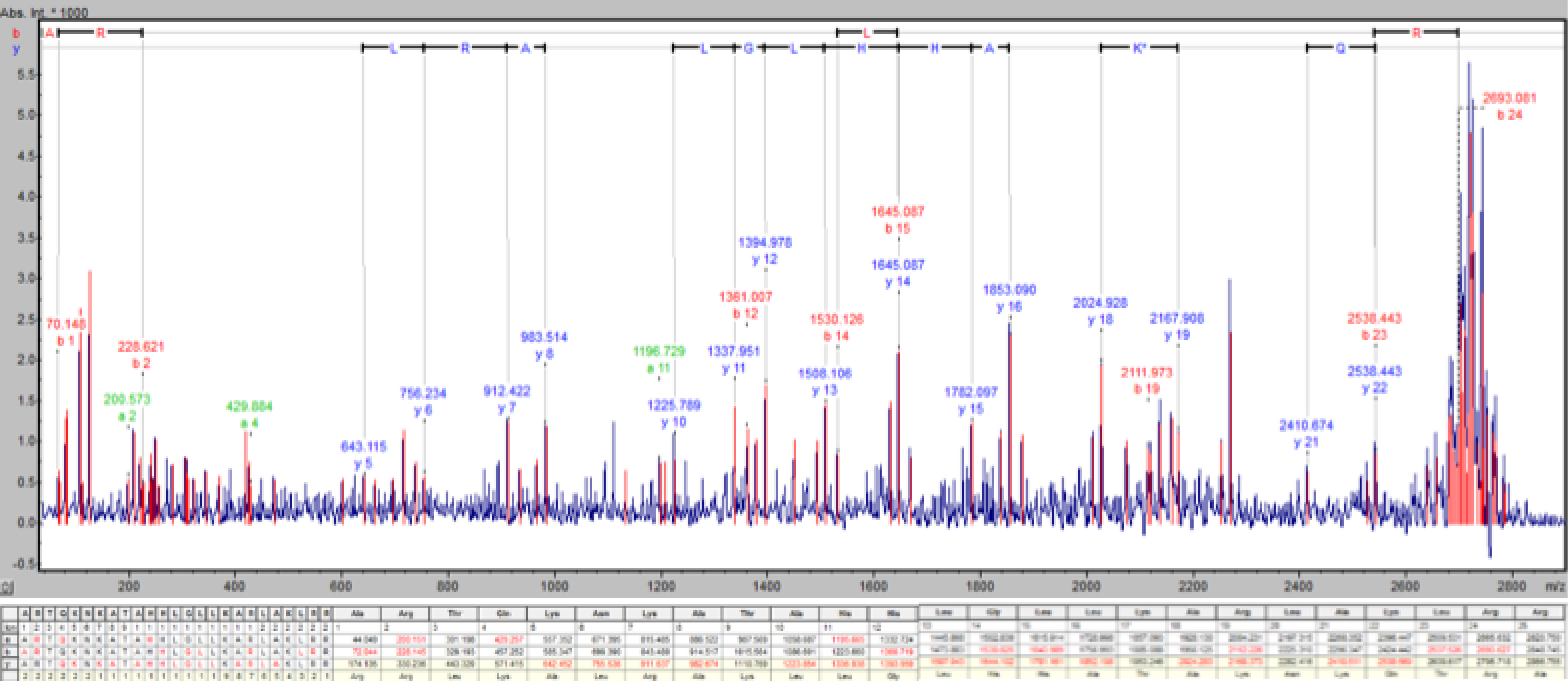 |
|  |
| **b.** |
| 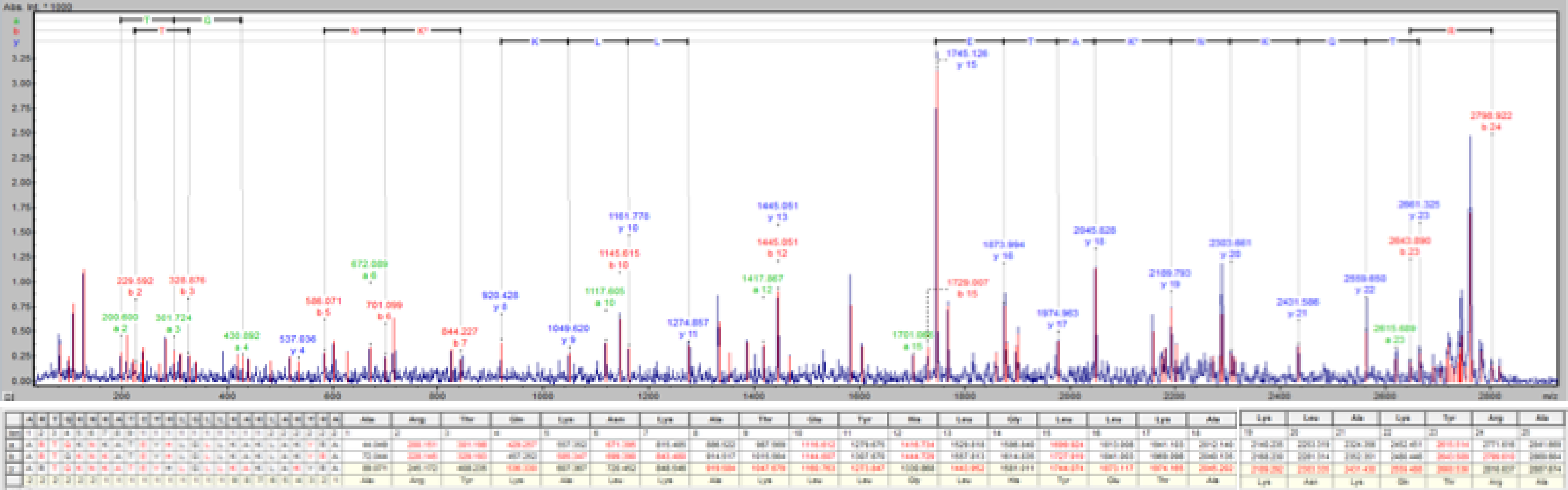 |
| **c.** |
| 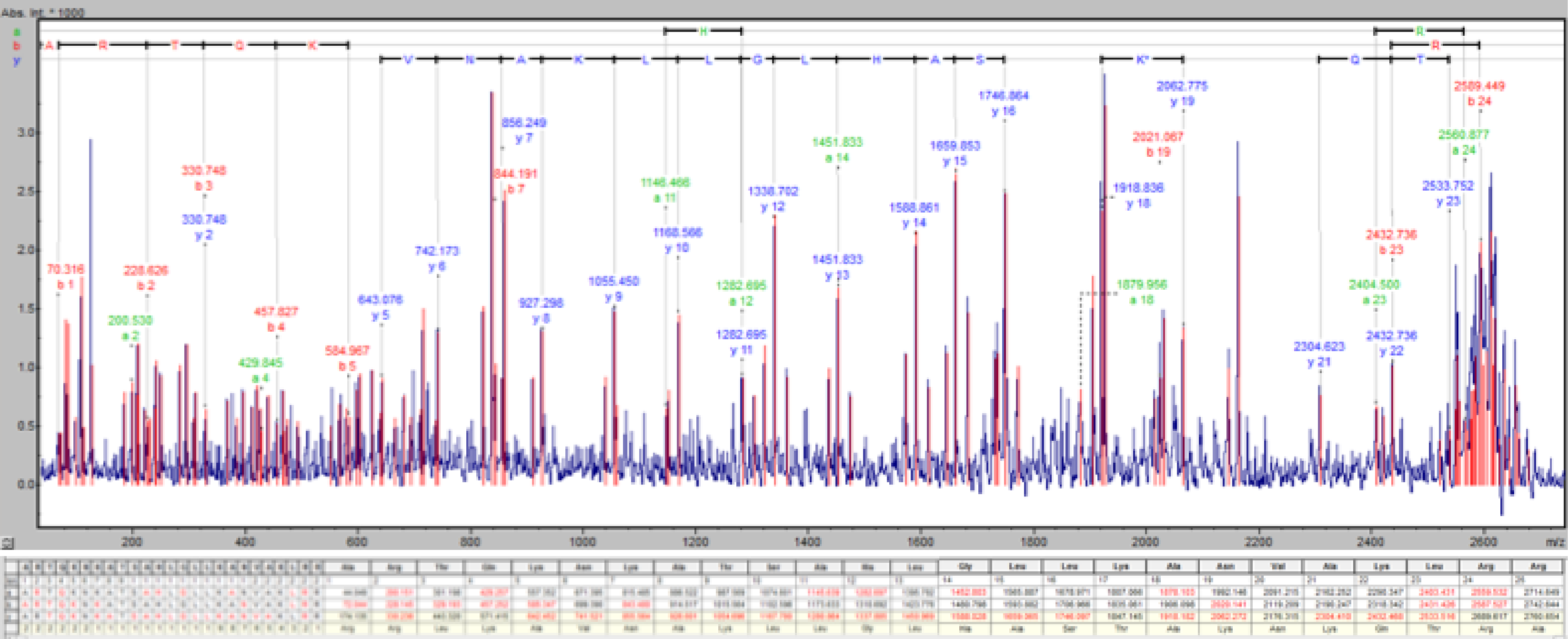 |

**Figure S3. MS/MS analyses of dmJMJD7 catalysed reaction products.** MS/MS spectra of (**a**) hydroxylated DRG1 peptide (parent peak at m/z = 2868.43), (**b**) dmJMJD7 hydroxylated DRG2 peptide (parent peak at m/z = 2889.59) and (**c**) dmJMJD7 hydroxylated GBP peptide (parent peak at m/z = 2762.39). The MS/MS spectra show the b ions stemming from the N-terminus and y ions from the C-terminus. After an initial MALDI-TOF MS was acquired on the reaction product, the hydroxylated peptide peak was selected as parent peak, which was then used for the tandem MS. The MS/MS data provide evidence that hydroxylation occurs at K22 in DRG1 and GBP, and at K21 in DRG2.


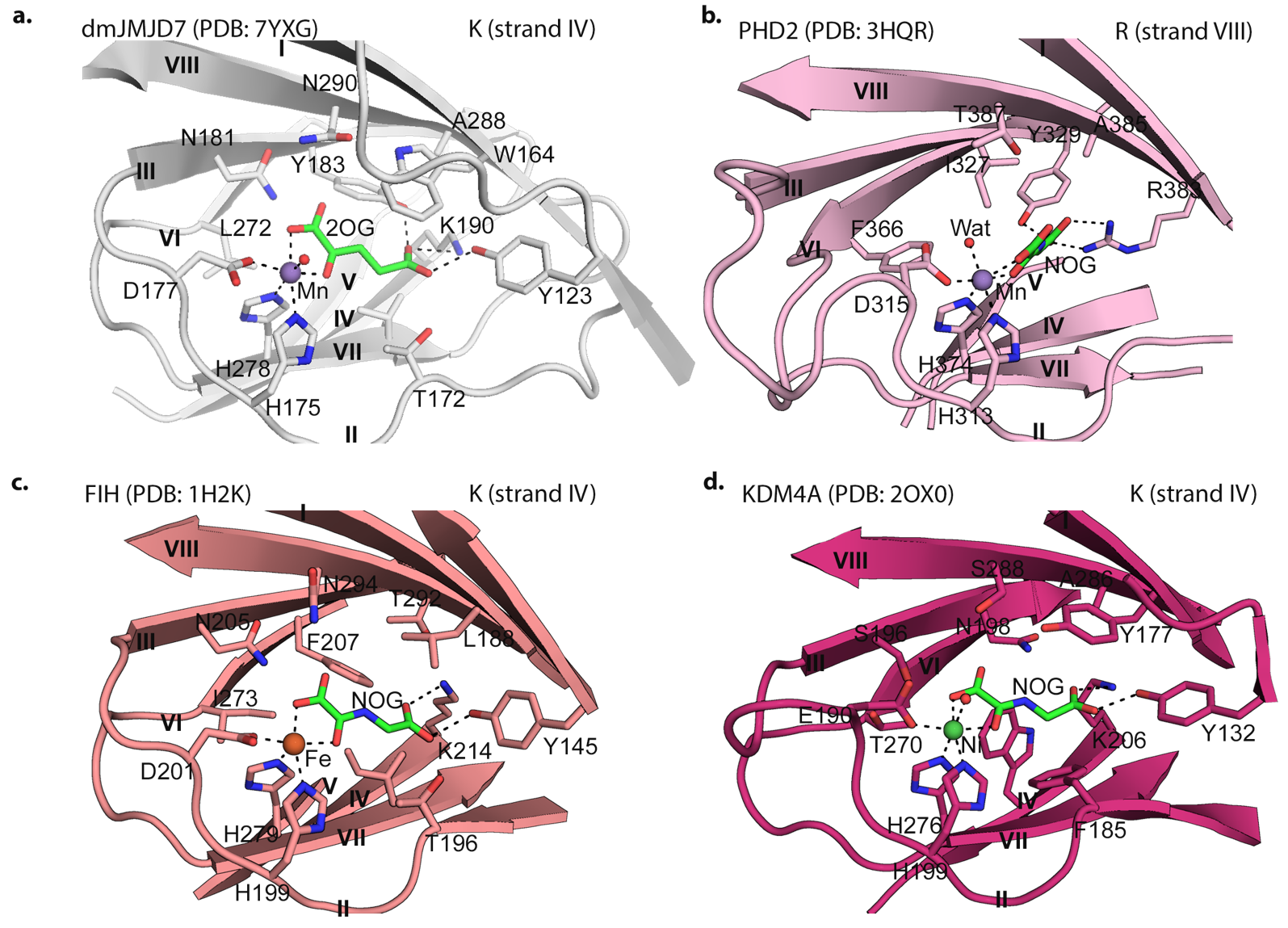


### **Figure S4. 2OG Comparison of 2OG/co-substrate binding by dmJMJD7 and representative JmjC (FIH and KDM4A) and non-JmjC (PHD2) 2OG oxygenases.** The metal ion is octahedrally coordinated by residues of the HxD/E..H motif, 2OG (co-substrate)/ NOG (a near 2OG isostere/ non-reactive inhibitor) and, usually, a water molecule. Binding of the 2OG C5-carboyxlate involves electrostatic interactions with a basic residue (R or K located either on DSBH strand-IV or VIII, as indicated; K190 for dmJMJD7), and often polar residues including alcohols, i.e. a Tyr located on a non-DSBH β-strand (Y123 and Y183 for JMJD7).

###
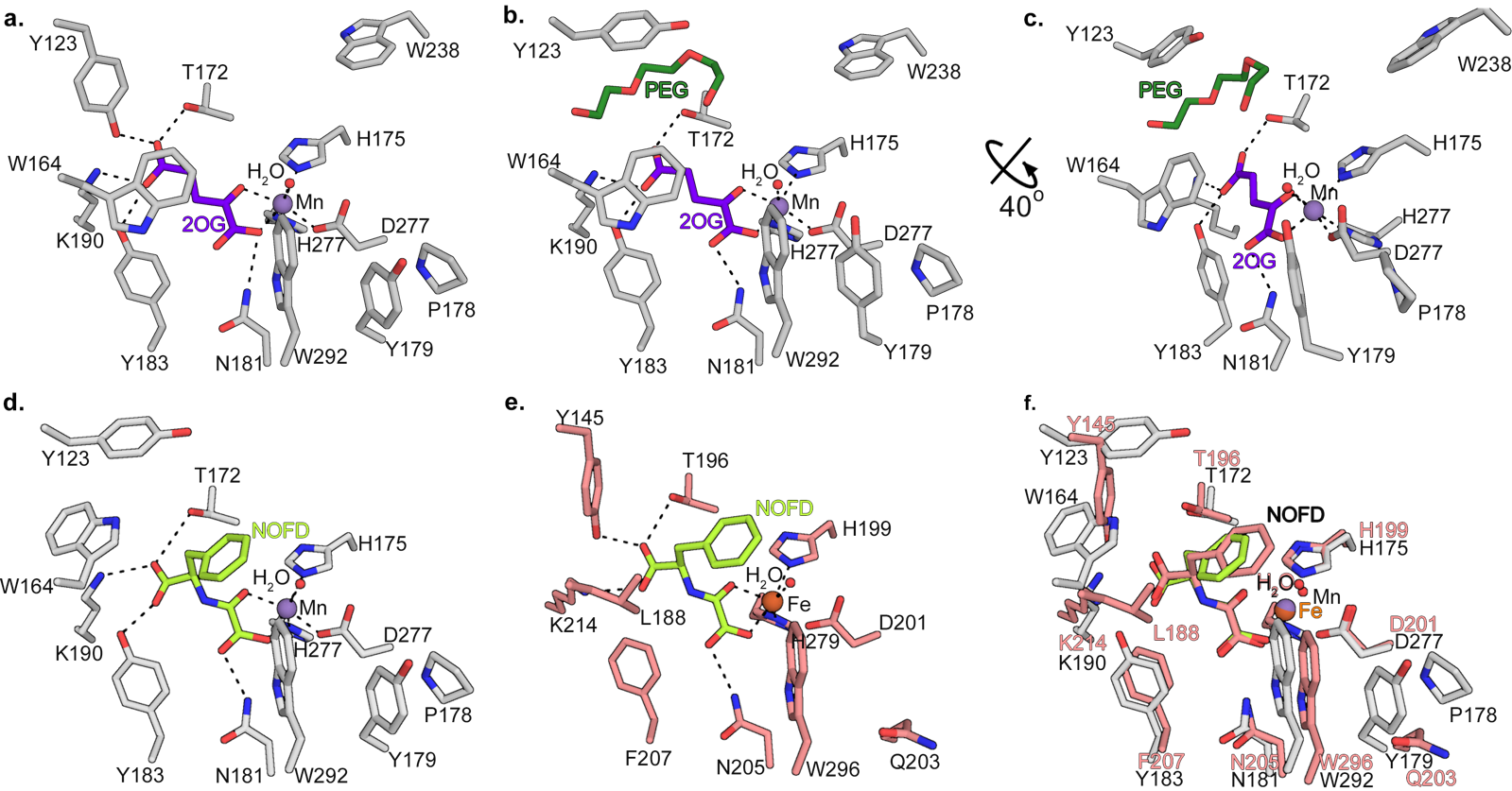


### **Figure S5. Comparison of active site views from (a) dmJMJD7.Mn^II^.2OG, (b, c) dmJMJD7.Mn^II^.2OG.PEG, (d) dmJMJD7.Mn^II^.NOFD, (e) FIH.Fe^II^.NOFD complex structures; (f) overlay of (d) and (e).**

**Table S1. Data collection and refinement statistics of dmJMJD7.ligand complexes.**

|  | dmJMJD7. Mn(II).2OG | dmJMJD7. Mn(II).Succinate | dmJMJD7. Mn(II).NOG | dmJMJD7. Mn(II).2,4-PDCA | dmJMJD7. Mn(II).NODA | dmJMJD7. Mn(II).NOFD |
| --- | --- | --- | --- | --- | --- | --- |
|  |  |  |  |  |  |  |
| **PDB codes** | 7YXG | 7YXH | 7YXI | 7YXJ | 7YXK | 7YXL |
| **Data collection** |  |  |  |  |  |  |
| Beamline (Wavelength, Å) | DLS I03 (0.9795) | DLS I24 (0.9686) | DLS I04 (0.9795) | DLS I24  (0.9686) | DLS I04 (0.9795) | DLS I04 (0.9795) |
| Detector | pilatus3 6m | pilatus3 6m | pilatus3 6m | pilatus3 6m | pilatus3 6m | pilatus3 6m |
| No. of crystals | 1 | 1 | 1 | 1 | 1 | 1 |
| Data processing | HKL2000[^1^](#_ENREF_1) | HKL2000[^1^](#_ENREF_1) | HKL2000[^1^](#_ENREF_1) | HKL2000[^1^](#_ENREF_1) | HKL2000[^1^](#_ENREF_1) | HKL2000[^1^](#_ENREF_1) |
| Space group | *P*2_1_ | *P*2_1_ | *P*2_1_ | *P*2_1_ | *P*2_1_ | *P*2_1_ |
| Cell dimensions |  |  |  |  |  |  |
| *a*, *b*, *c* (Å) | 73.33, 62.86, 99.00 | 54.63, 65.33, 103.67 | 55.67, 64.89, 103.08 | 53.56, 65.76, 206.96 | 55.36, 64.53, 99.49 | 55.02, 65.16, 99.22 |
| *α*, *β*, *γ* (°) | 90, 109.07, 90 | 90, 101.73, 90 | 90, 102.55, 90 | 90, 97.78, 90 | 90, 100.93, 90 | 90, 100.77, 90 |
| No. of molecules/ ASU | 2 | 2 | 2 | 4 | 2 | 2 |
| No. reflections | 103935 (6886)* | 31994 (1067)* | 46554 (3429)* | 52754 (1740)* | 26179 (3795)* | 35134 (2552)* |
| Resolution (Å) | 45.89-1.64 (1.68-1.64)* | 65.33-2.30 (2.42-2.30)* | 54.31-2.03 (2.08-2.03)* | 62.62-2.45 (2.58-2.45)* | 54.35-2.43 (2.56-2.43)* | 40.42-2.20 (2.26-2.20)* |
| *R*_sym_ or *R*_merge_ | 0.076 (1.369)* | 0.114 (1.052)* | 0.095 (0.768)* | 0.125 (0.958)* | 0.173 (0.900)* | 0.121 (1.080)* |
| *R*_pim_ | 0.041 (0.808)* | 0.056 (0.512)* | 0.073 (0.596)* | 0.062 (0.466)* | 0.084 (0.443)* | 0.067 (0.582)* |
| *I*/σ*I* | 18.0 (1.2)* | 8.3 (1.6)* | 9.5 (2.0)* | 7.8 (1.7)* | 8.1 (1.7)* | 9.9 (1.2)* |
| CC (1/2) | 0.996 (0.580)* | 0.996 (0.529)* | 0.996 (0.564)* | 0.995 (0.552)* | 0.991 (0.692)* | 0.995 (0.559)* |
| Completeness (%) | 99.8 (99.9)* | 99.9 (100)* | 100 (99.8)* | 99.8 (100)* | 100 (100)* | 99.8 (99.7)* |
| Redundancy | 4.8 (4.8)* | 5.0 (4.5)* | 5.0 (4.7)* | 4.9 (5.0)* | 5.1 (5.0)* | 5.1 (5.2)* |
|  |  |  |  |  |  |  |
| **Refinement** | phenix[^2^](#_ENREF_2) | phenix[^2^](#_ENREF_2) | phenix[^2^](#_ENREF_2) | phenix[^2^](#_ENREF_2) | phenix[^2^](#_ENREF_2) | phenix[^2^](#_ENREF_2) |
| *R*_work/_ *R*_free_^‡^ | 0.165/0.185 | 0.182/0.227 | 0.188/0.207 | 0.207/0.229 | 0.211/0.254 | 0.201/0.235 |
| R.m.s deviations |  |  |  |  |  |  |
| -Bond lengths (Å) | 0.008 | 0.011 | 0.005 | 0.006 | 0.005 | 0.005 |
| -Bond angles (º) | 0.935 | 1.330 | 1.012 | 1.101 | 0.737 | 1.078 |
|  |  |  |  |  |  |  |

*Highest resolution shell shown in parentheses.

**R_sym_ = ∑|*I*-<*I*>|/∑*I*, where *I* is the intensity of an individual measurement and <*I*> is the average intensity from multiple observations.

^‡^R_factor_ = ∑*_hkl_*||*F*_obs_(*hkl*)| − k |*F_c_*_alc_(*hkl*)||/ ∑*_hkl_*|*F*_obs_(*hkl*)| for the working set of reflections; R_free_ is the R_factor_ for ~5% of the reflections excluded from refinement.

**Supplementary References**

1 Otwinowski, Z. & Minor, W. Processing of X-ray diffraction data collected in oscillation mode. *Methods in Enzymology* **276**, 307-326, doi:10.1016/S0076-6879(97)76066-X (1997).

2 Adams, P. D. *et al.* PHENIX : a comprehensive Python-based system for macromolecular structure solution. *Acta Crystallographica D Biological Crystallography* **66**, 213-221, doi:10.1107/S0907444909052925 (2010).
